# Establishment and validation of a prediction model for apnea on bronchiolitis

**DOI:** 10.3389/fped.2024.1397750

**Published:** 2024-07-10

**Authors:** Qiuyan Xu, Li Shen, Min Lu, Shuangqin Ran, Wujun Jiang, Jun Hua, Linlin Li

**Affiliations:** ^1^ Department of Pediatrics, Suzhou Research Center of Medical School, Suzhou Hospital, Affiliated Hospital of Medical School, Nanjing University, Suzhou, China; ^2^ Department of Pharmacy, Suzhou Research Center of Medical School, Suzhou Hospital, Affiliated Hospital of Medical School, Nanjing University, Suzhou, China; ^3^ Department of Respiratory Medicine, Children’s Hospital of Soochow University, Suzhou, China; ^4^ Department of Respiratory Medicine, Children’s Hospital of Wujiang District, Children’s Hospital of Soochow University, Suzhou, China; ^5^ Department of Respiratory Medicine, Children’s Hospital of Nanjing Medical University, Nanjing, China

**Keywords:** bronchiolitis, apnea, nomogram, predictive model, risk

## Abstract

**Objective:**

The objective of this study is to examine the risk factors associated with apnea in hospitalized patients diagnosed with bronchiolitis and to develop a nomogram prediction model for the early identification of patients who are at risk of developing apnea.

**Methods:**

The clinical data of patients diagnosed with acute bronchiolitis and hospitalized at the Children's Hospital of Nanjing Medical University between February 2018 and May 2021 were retrospectively analyzed. LASSO regression and logistic regression analysis were used to determine the risk factors for apnea in these patients. A nomogram was constructed based on variables selected through multivariable logistic regression analysis. Receiver operating characteristic (ROC) curve and calibration curve were used to assess the accuracy and discriminative ability of the nomogram model, and decision curve analysis (DCA) was performed to evaluate the model's performance and clinical effectiveness.

**Results:**

A retrospective analysis was conducted on 613 children hospitalized with bronchiolitis, among whom 53 (8.6%) experienced apnea. The results of Lasso regression and Logistic regression analyses showed that underlying diseases, feeding difficulties, tachypnea, WBC count, and lung consolidation were independent risk factors for apnea. A nomogram prediction model was constructed based on the five predictors mentioned above. After internal validation, the nomogram model demonstrated an AUC of 0.969 (95% CI 0.951–0.987), indicating strong predictive performance for apnea in bronchiolitis. Calibration curve analysis confirmed that the nomogram prediction model had good calibration, and the clinical decision curve analysis (DCA) indicated that the nomogram was clinically useful in estimating the net benefit to patients.

**Conclusion:**

In this study, a nomogram model was developed to predict the risk of apnea in hospitalized children with bronchiolitis. The model showed good predictive performance and clinical applicability, allowing for timely identification and intensified monitoring and treatment of high-risk patients to improve overall clinical prognosis.

## Introduction

Bronchiolitis, a common respiratory infection affecting infants and young children, is a significant reason for hospitalization worldwide in the initial year of life (1). Although many cases are mild, some children may develop severe symptoms like apnea, tachypnea, cyanosis, dyspnea, or severe hypoxia, leading to hospitalization. In critical situations, children may need to be transferred to the Pediatric Intensive Care Unit (PICU) for advanced life-sustaining interventions or, tragically, may succumb to the illness (2).

Apnea poses a life-threatening risk in bronchiolitis patients, frequently necessitating admission to the PICU for critical interventions (3, 4). Approximately 20% of bronchiolitis patients with respiratory syncytial virus (RSV) infection who require intensive care support may manifest apnea (5). The presence of apnea in bronchiolitis patients is often indicative of a poor prognosis with a heightened mortality risk. Some studies recommend immediate hospitalization for bronchiolitis patients at apnea risk, with some cases warranting ongoing monitoring and treatment in the PICU (6–8).

The nomogram, a visual statistical model, offers an intuitive representation and quantification of disease risk, aiding in early disease detection and treatment by clinical staff. Currently, there is a lack of nomogram-based risk assessment for predicting in-hospital apnea in bronchiolitis patients. This study aims to summarize the clinical characteristics of apnea in patients with bronchiolitis, identify the risk factors associated with apnea in bronchiolitis, and develop a personalized prediction model. This model will present clinicians with a reliable tool to identify high-risk patients and implement appropriate monitoring and treatment strategies to improve clinical outcomes.

## Methods

### Study design and populations

Patients diagnosed with acute bronchiolitis and admitted to Children's Hospital of Nanjing Medical University from February 2018 to May 2021 were enrolled in this study. Inclusion criteria required patients to meet the diagnostic criteria for acute bronchiolitis (9, 10) and be aged ≤2 years. Patients with incomplete medical data, nosocomial infections, or wheezing attributed to factors like tuberculosis or non-infectious causes (e.g., bronchial foreign bodies) were excluded from the study. The study was approved by the Ethics Committee of the Children's Hospital of Nanjing Medical University (ethics number: 202206125-1), and informed consent was obtained from the parents of all participating children.

### Data collection

Data for patients on general characteristics, past and personal medical history, clinical presentation, laboratory findings, pathogens, and pulmonary Imaging were accurately recorded. General characteristics included age, gender, and disease duration pre-admission. Past and personal medical history, including prematurity, congenital heart disease (CHD), bronchopulmonary dysplasia (BPD), history of apnea, immunodeficiency, and severe malnutrition, were classified as underlying diseases. White blood cell (WBC) count and C-reactive protein (CRP) levels were observed in the laboratory findings. Clinical manifestations included fever, cough, vomiting, feeding difficulties, apnea, tachypnea, retractions, as well as lung crackles.

The pathogens comprised Mycoplasma pneumoniae (MP), RSV, influenza viruses A and B (IVA, IVB), as well as parainfluenza viruses 1, 2, and 3 (PIV1, PIV2, PIV3), along with adenovirus (ADV). Pulmonary imaging encompassed findings of pulmonary consolidation, pleural effusion, and pulmonary atelectasis.

### Statistical analysis

Continuous variables were presented as median (interquartile range) and assessed for group differences using the Mann-Whitney *U* test. Categorical variables were expressed as counts and proportions, with group disparities examined through either the Pearson chi-square test or Fisher's exact test.

LASSO regression was applied for variable selection in high-dimensional data through 10-fold cross-validation to ascertain the optimal Lambda parameters. The Lambda.1se value with the smallest validation error was determined as the optimal solution for variable screening. The selection of preliminary screening variables was determined by nonzero coefficients in the LASSO regression model. Following this, a nomogram model was established for early prediction of apnea in hospitalized patients with acute bronchiolitis through the results of multiple logistic regression analysis.

The area under the ROC curve (AUC) was utilized to evaluate the predictive performance of the nomogram model. Internal validation of the nomogram was conducted through 1,000 iterations of Bootstrap resampling, while a calibration curve was employed to assess predictive consistency. Additionally, the Hosmer-Lemeshow test was conducted. To calculate the net benefit at each risk threshold probability of the model, Decision Curve Analysis (DCA) was used to confirm the clinical application value of the nomogram model.

Statistical analysis and graphics were conducted using R 4.1.1(https://www.r-project.org/), The “*rms*”, “*Hmisc*”*,* “*glmnet*”, “*ResourceSelection*”, *and* “*rmda*” R packages were utilized, and two-sided *P* < 0.05 was considered statistically significant.

## Results

### Clinical characteristics

A total of 613 hospitalized patients diagnosed with bronchiolitis from February 2018 to May 2021 were included in this study after excluding 22 individuals with incomplete clinical data. The incidence of apnea among the patients was 8.6%. The median age was 4.00 (2.30, 7.83) months, with 40 (6.5%) patients having underlying diseases. The primary clinical manifestations observed in bronchiolitis patients were fever (29.4%), cough (99.7%), tachypnea (21.9%), apnea (8.6%), retractions (9.5%), vomiting (17.3%), and feeding difficulties (19.4%). RSV was the most common pathogen (43.1%), followed by MP (15.7%). Pulmonary imaging revealed segmental pulmonary consolidation in 8% of patients, pleural effusion in 0.2%, and pulmonary atelectasis in 1.8% of patients (Table 1).

**Table 1:** Clinical data and comparison of patients with apnea and non-apnea.

	Overall (*n* = 613)	Apnea group (*n* = 53)	Non-apnea group (*n* = 560)	*P*
General characteristics				
Age (month) (IQR)	4.00 (2.30, 7.83)	3.17 (1.87, 4.63)	4.34 (2.33, 8.00)	0.003
Age <6 months (%)	399 (65.1）	45 (84.9）	354 (63.2）	<0.001
Gender, male (%)	436 (71.1)	31 (58.5)	405 (72.3)	0.214
Disease duration pre-admission (day) (IQR)	6.00 (4.00, 13.00)	4.00 (3.00, 8.00)	6.00 (4.1, 14.00)	<0.001
Hospitalization days (day) (IQR)	9.00 (7.00, 15.00)	19.00 (14.00, 28.00)	8.00 (7.00, 10.00)	<0.001
Past and personal medical history				
Underlying diseases (%)	40 (6.5)	22 (41.5)	18 (3.2)	<0.001
Preterm birth (%)	8 (1.3)	5 (9.4)	3 (0.5)	
BPD (%)	8 (1.3)	7 (13.2)	1 (0.2)	
CHD (%)	27 (4.4)	13 (24.5)	14 (2.5)	
Apnea history (%)	2 (0.3)	1 (1.9)	1 (0.2)	
Severe malnutrition (%)	1 (0.2)	1 (1.9)	0 (0.0)	
Immunodeficiency (%)	1 (0.2)	0 (0.0)	1 (0.2)	
Laboratory findings				
WBC count (×10^9^/L) (IQR)	9.32 (6.99, 12.33)	10.82 (7.38, 17.49)	9.26 (6.97, 12.13)	0.01
CRP levels (IQR)	0.82 (0.23, 3.22)	1.12 (0.10, 7.00)	0.76 (0.23, 3.01)	0.392
Clinical presentation				
Fever (%)	180 (29.4)	42 (79.2)	138 (24.6)	<0.001
Cough (%)	611 (99.7)	53 (100.0)	558 (99.6)	1
Vomiting (%)	106 (17.3)	12 (22.6）	94 (16.8)	0.281
Feeding difficulties (%)	119 (19.4)	43 (81.1)	76 (13.6)	<0.001
Tachypnea (%)	134 (21.9)	40 (75.5)	94 (16.8)	<0.001
Retractions (%)	58 (9.5)	24 (45.3)	34 (6.1)	<0.001
Crackles (%)	530 (86.5)	52 (98.1)	478 (85.4)	0.017
Pathogens				
MP (%)	96 (15.7)	8 (15.1)	88 (15.7)	1
RSV (%)	264 (43.1)	18 (34.0)	246 (43.9)	0.209
IVA (%)	2 (0.3)	0 (0.0)	2 (0.4)	1
IVB (%)	0 (0.0)	0 (0.0)	0 (0.0)	NA
PIV1 (%)	2 (0.3)	0 (0.0)	2 (0.4)	1
PIV3 (%)	54 (8.8)	6 (11.3)	48 (8.6)	0.673
ADV (%)	11 (1.8)	5 (9.4)	6 (1.1)	<0.001
Pulmonary imaging				
Pulmonary consolidation (%)	49 (8.0)	19 (35.8)	30 (5.4)	<0.001
Pleural effusion (%)	1 (0.2)	1 (1.9)	0 (0.0)	0.141
Pulmonary atelectasis (%)	11 (1.8)	7 (13.2)	4 (0.7)	<0.001

Characteristics were summarized as either median (IQR) or frequency (%). BPD, bronchopulmonary dysplasia; CHD, congenital heart disease; MP, mycoplasma pneumoniae; RSV, respiratory syncytial virus; IVA, influenza virus A; IVB, influenza virus B; PIV1, parainfluenza virus 1; PIV3, parainfluenza virus 3; ADV, adenovirus.

Apnea is defined as a complete cessation of oral and nasal airflow lasting at least ten seconds, including central apnea, obstructive apnea, and mixed apnea (11, 12). Patients were divided into the apnea group or the non-apnea group depending on whether apnea occurred during their hospitalization. The mean age of children in the apnea group was 3.17 (1.87, 4.63) months, significantly lower than that of the non-apnea group at 4.34 (2.33, 8.00) months. Patients with apnea had a significantly shorter duration of illness before admission compared to non-apnea patients. However, the mean hospitalization duration was significantly longer for the apnea group at 19 (14.00, 28.00) days. The occurrence of underlying diseases in the apnea group was significantly higher than in the non-apnea group (41.5% vs. 3.2%). Children with apnea exhibited more severe clinical symptoms, including higher incidences of fever (79.2% vs. 24.6%), feeding difficulties (81.1% vs. 13.6%), tachypnea (75.5% vs. 16.8%), retractions (45.3% vs. 6.1%), crackles (98.1% vs. 85.4%). Radiographically, they were more likely to present with pulmonary consolidation (35.8% vs. 5.4%) and pulmonary atelectasis (13.2% vs. 0.7%). Moreover, the percentage of apnea patients with ADV was higher than that of non-apnea patients (9.4% vs. 1.1%), and all *p*-values were less than 0.05 (Table 1).

### Risk factors for apnea

**Figure 1:**
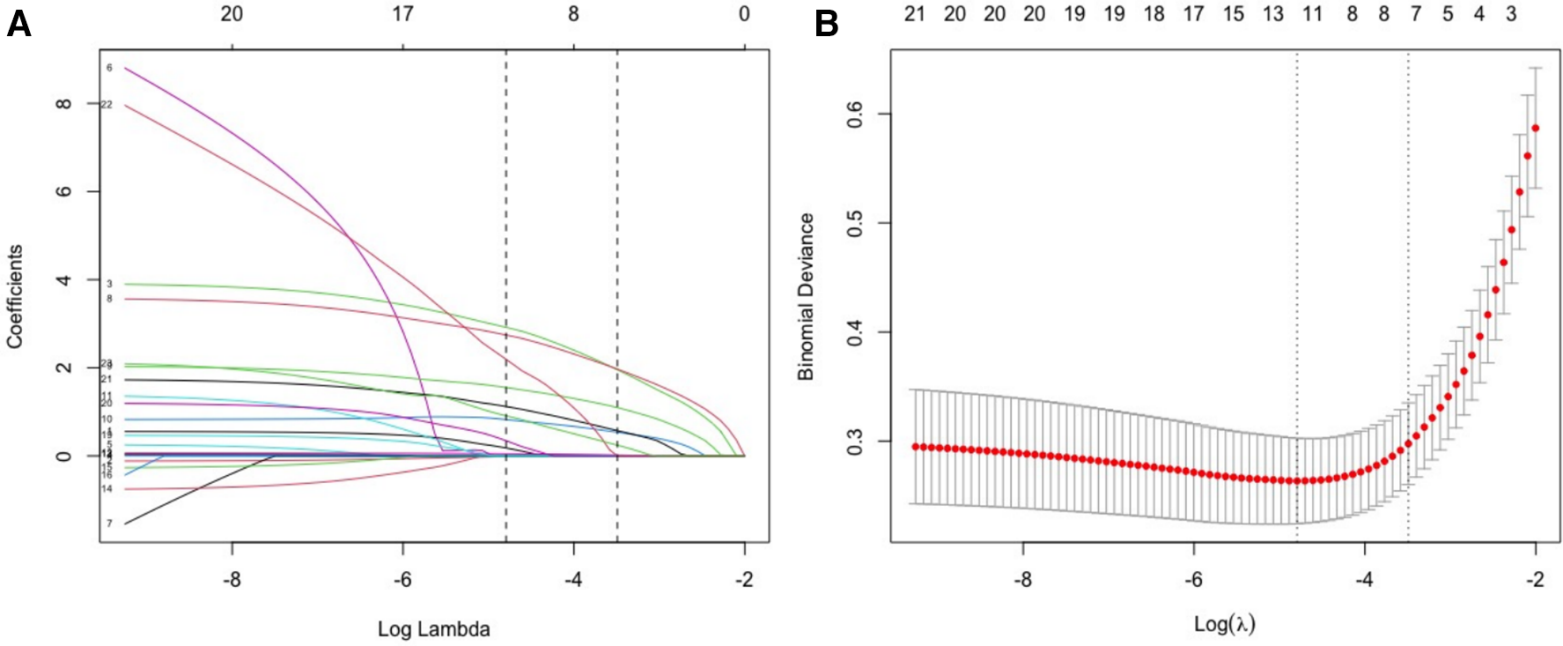
Screening of variables of possible risk factors for apnea based on LASSO regression. (**A**) The variation characteristics of the LASSO coefficient of variables; (**B**) the selection process of the Optimal parameter (*λ*) in the LASSO model used cross-validation via minimum criteria.

Except for the length of hospitalization, seven potential predictors, including underlying diseases, feeding difficulties, tachypnea, retractions, white blood cell count (WBC), pulmonary consolidation, and pulmonary atelectasis, were identified through LASSO regression from the other 24 variables (Figure 1). Univariate and multifactorial logistic regression analyses confirmed that underlying diseases, feeding difficulties, tachypnea, WBC count, and pulmonary consolidation were independent risk factors for apnea in hospitalized patients with acute bronchiolitis (Table 2).

**Table 2:** Factors associated with apnea in univariable and multivariable analyses.

Variable	Univariate logistic regression		Multifactorial logistic regression	
	OR (95% CI)	*p*	OR (95% CI)	*p*
Underlying diseases	21.369 (10.461–44.507)	<0.001	41.491 (13.038–155.113)	<**0.001**
Feeding difficulties	27.384 (13.719–59.830)	<0.001	36.595 (13.090–126.006）	<**0**.**001**
Tachypnea	15.254 (8.0576–30.693)	<0.001	7.533 (2.824–21.439)	<**0**.**001**
Retractions	12.803 (6.730–24.445）	<0.001	2.547 (0.919–7.034)	0.070
WBC count	1.080 (1.046–1.117)	<0.001	1.074 (1.021–1.129)	**0**.**005**
Pulmonary consolidation	9.873 (5.007–19.290)	<0.001	5.021 (1.467–17.158)	**0**.**009**
Pulmonary atelectasis	21.152 (6.160–83.262）	<0.001	8.449 (0.318–266.926)	0.296

OR, odds ratio.

Bolded values indicate statistical significance (*p* < 0.05).

### Nomogram model establishment

**Figure 2:**
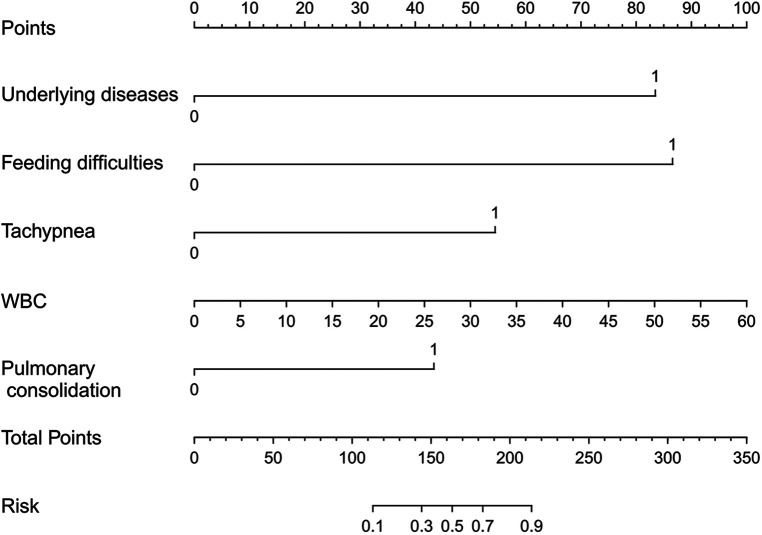
Nomogram used to predict possible risk factors for apnea in patients with bronchiolitis. 1 indicates the presence of a certain change, while 0 indicates the absence of a certain change.

A nomogram prediction model was established to predict the likelihood of apnea in bronchiolitis, utilizing five independent risk factors that exhibited statistical significance (*P* < 0.05) in multivariate logistic regression analysis (Figure 2). The nomogram interpretation method involved drawing a vertical line on the horizontal axis of each predictor variable based on a specific score on the “Points” scale. Subsequently, the scores from all five predictor variables were aggregated to determine the total score. The vertical line drawn at this total score intersected with the horizontal axis of the “Risk” scale, representing the predicted risk value. For example, if a patient with bronchiolitis has underlying diseases, feeding difficulties, no tachypnea, a WBC count of 10 × 109/L, and no pulmonary consolidation on imaging, the patient's corresponding total score would be 83 + 88 + 0 + 16 + 0 = 187 points. The predictive value of the nomogram is about 65%, indicating that the patient has a 65% likelihood of experiencing apnea.

### Internal validation of the nomogram model

**Figure 3:**
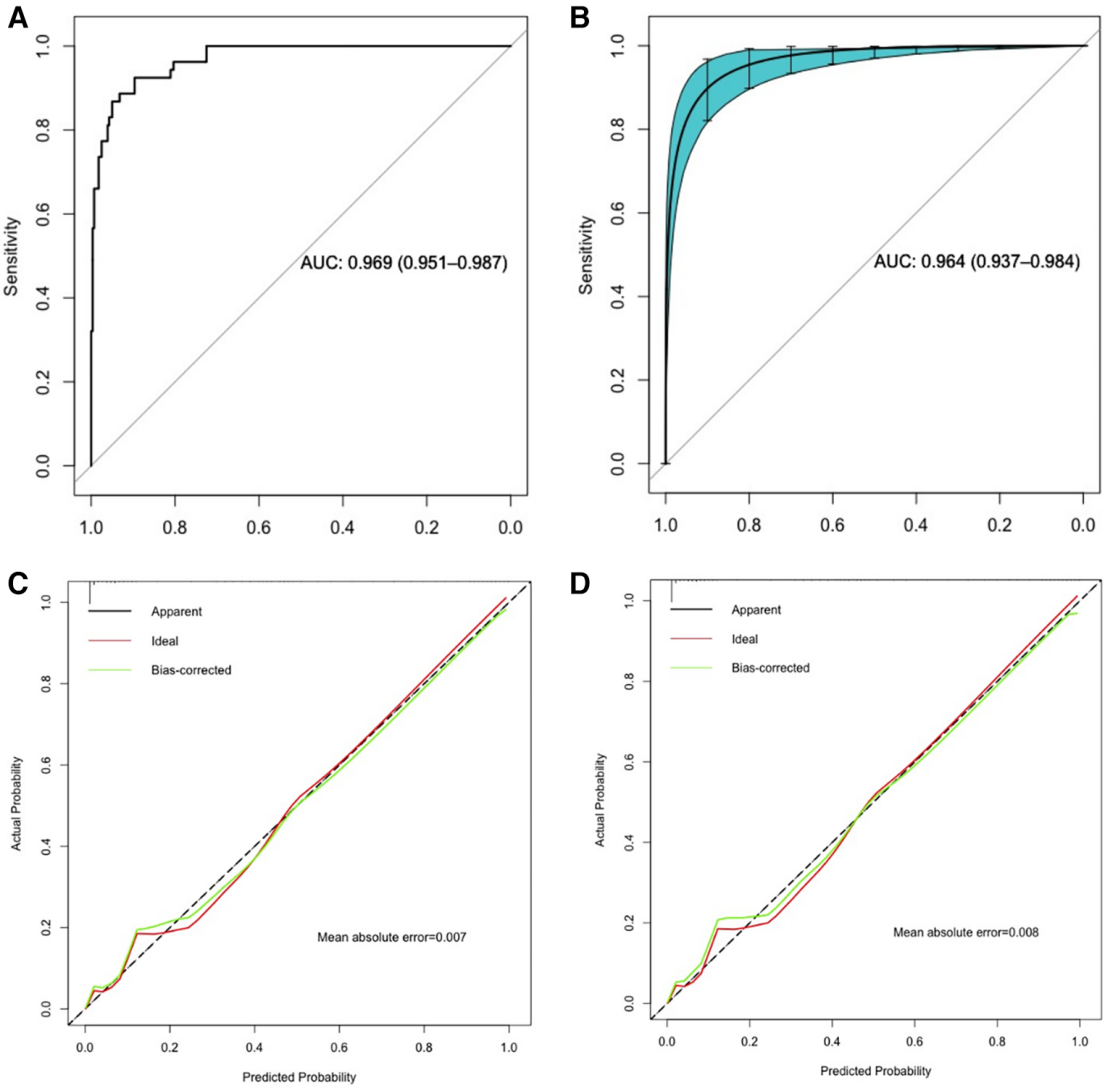
ROC curve and calibration curve analysis of the nomogram predictive model. (**A**,**C**): ROC curve and calibration curve of the predictive model; (**B**,**D**): ROC curve and calibration curve of the predictive model with internal validation using 1,000 bootstrap resamples.

The predictive model predicted the risk of apnea occurrence with an AUC greater than all single predictor variables, with an AUC of 0.969 (95% confidence interval: 0.951–0.987) (Table 3). Bootstrap resampling was used 1,000 times to internally validate the nomogram. The results showed that the AUC value of the nomogram model after internal validation was 0.964 (95% confidence interval: 0.937–0.984), indicating that the nomogram model has good discriminatory ability. The results of the Hosmer-Lemeshow test confirmed that there was no significant difference in the prediction deviation between the risk prediction value of the nomogram model and the actual observed value (*p* = 0.977). Additionally, the calibration curve showed that the nomogram model exhibited good calibration and consistent results before and after internal validation (Figure 3).

**Table 3:** AUC and 95% CI for the nomogram and model variables.

Variable	AUC	95% CI
Nomogram	0.969	0.951–0.987
Underlying diseases	0.692	0.624–0.759
Feeding difficulties	0.838	0.783–0.892
Tachypnea	0.793	0.733–0.854
WBC count	0.607	0.515–0.700
Pulmonary consolidation	0.653	0.587–0.718

### Clinical applicability of the nomogram model

**Figure 4:**
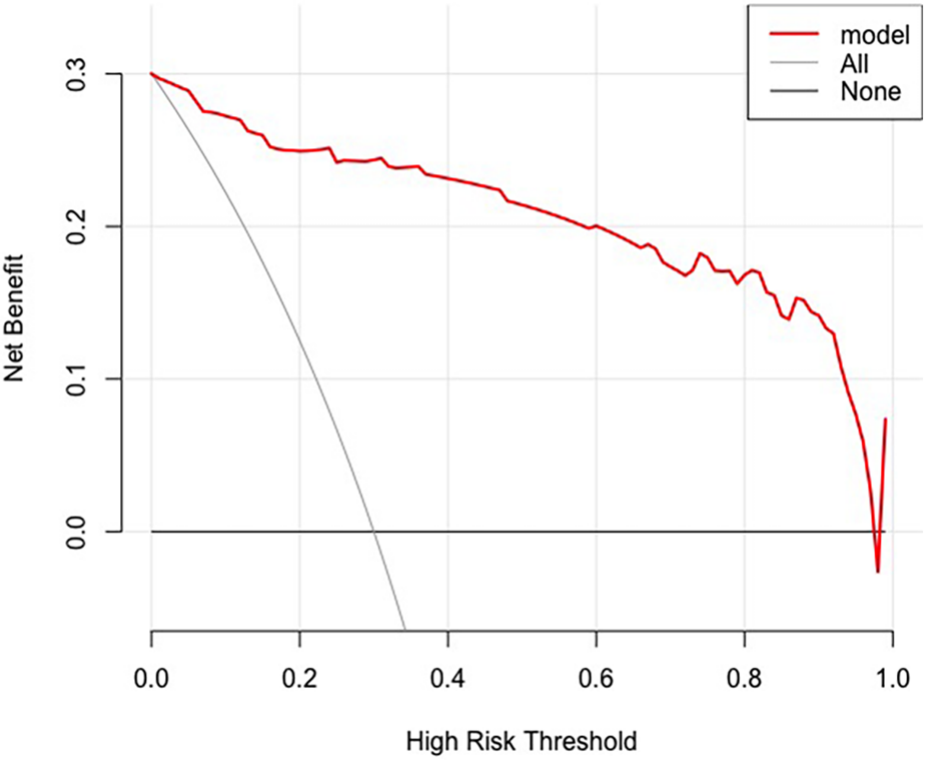
Decision curve analysis curve of the nomogram predictive model.

Clinical decision curve analysis (DCA) was used to evaluate the clinical utility of the nomogram prediction model (Figure 4). It can be seen from the DCA that when the threshold probability was between 0.00 and 0.97, the clinical net benefits of using this nomogram model were greater than those of the “all-intervention” and “no-intervention” strategies. This indicates that the nomogram prediction model performed well and had good clinical applicability.

## Discussion

Acute bronchiolitis is an acute respiratory infectious disease mainly caused by viral infection, posing a significant global health burden, especially in high-risk infants and young children (13). Apnea, a critical complication of bronchiolitis, can pose a life-threatening risk, especially in infants under 6 weeks old, where it may manifest as the sole clinical symptom without other typical features of bronchiolitis (14, 15). The results of this study demonstrate that the incidence of apnea in hospitalized patients with bronchiolitis is 8.6%, which is consistent with the previously reported incidence of 1.2%–28.8% (3, 6, 7, 15, 16). This suggests a higher incidence of apnea in patients with bronchiolitis. Given the high incidence and associated poor clinical prognosis, prompt evaluation and integration of risk factors for apnea in bronchiolitis patients, along with screening of high-risk individuals, can help clinicians develop precise prevention and management strategies. This is important for improving patient prognosis and reducing the risk of mortality. Therefore, we developed a nomogram prediction model to predict the risk of apnea in patients with bronchiolitis. The discriminative power (AUC) of the nomogram prediction model, based on feeding difficulties, underlying diseases, tachypnea, WBC, and pulmonary consolidation to predict the risk of apnea was 0.969 (95% CI, 0.951–0.987). This shows good discriminative performance and clinical applicability, with substantial consistency between predicted and actual probabilities.

So far, several studies have identified predictors of apnea in bronchiolitis patients (3, 6, 7). Ramos Fernández et al. identified fever, cesarean section, low body weight, a history of apnea and severe bacterial infections as significant predictors of apnea in children diagnosed with bronchiolitis (6). Schroeder AR et al. showed that children were deemed high-risk for apnea if they were younger, premature, had a parental report of apnea, had low or high respiratory rates, or had low oxygen saturation (3). In our study, the underlying diseases identified as risk factors were generally in agreement with those reported in the above studies. The risk of apnea is significantly increased in patients with underlying diseases such as premature birth, CHD, BPD, a history of apnea, severe malnutrition, or immunodeficiency. Previous research has identified feeding difficulties as an important clinical manifestation in severe bronchiolitis in younger infants (17), while tachypnea and low oxygen saturation have been recognized as important manifestations of severe bronchiolitis (18). In the present study, we revealed that feeding difficulties and tachypnea are suggestive of a more severe clinical presentation and serve as important predictors of apnea in patients.

Pulmonary imaging findings in bronchiolitis often present nonspecific features, such as interstitial pulmonary inflammation, pulmonary hyperventilation, peribronchial inflammatory lesions, diffuse inflammatory infiltration, and occasionally pulmonary parenchymal changes with associated atelectasis (19). Currently, the impact of pulmonary imaging changes on capillary bronchiolitis remains underreported, with no documented instances of pulmonary solid lesions being identified as a risk factor for apnea. In this study, 8% of bronchiolitis patients developed segmental pulmonary consolidation, and the results suggest that segmental pulmonary consolidation is a risk factor for apnea. Segmental pulmonary consolidation in infants and young children may indicate a more severe inflammatory response. However, due to the lack of routine pulmonary imaging examinations for bronchiolitis, further research is needed to explore the prevalence and significance of pulmonary consolidation in relation to apnea.

RSV is the predominant viral pathogen associated with bronchiolitis, particularly in severe cases among infants (6), as supported by our findings. In developing countries, RSV contributes significantly to morbidity and mortality (1). Previous studies have indicated that RSV-related apnea occurs in 16%–20% of cases (8). Our study revealed that 34% of patients with apnea were RSV-positive, but this did not significantly differ from those without apnea. Contrary to expectations, RSV infection does not appear to be a risk factor for apnea in bronchiolitis, in line with the conclusions drawn by Schroeder et al. (3).

An elevated WBC count is commonly indicative of bacterial infection. Previous studies have confirmed that bacterial infection in bronchiolitis is often related to the severity of the disease (20–24). K Thorburn et al. conducted an etiological examination on 165 patients with severe RSV bronchiolitis who required admission to the PICU. They found that the incidence of bacterial co-infection in the lungs was as high as 42%, and that bacterial infection often indicates that children with RSV bronchiolitis have a more severe degree of disease (23). Similarly, in a recent retrospective cohort study, the authors found that among children with bronchiolitis who were under invasive mechanical ventilation, 37.7% had a bacterial infection. This was associated with a prolonged PICU stay and mechanical ventilation time (20). Our results are similar to previous studies in that bacterial infection indicates more severe disease and is a risk factor for apnea in children with bronchiolitis.

Our study has several potential limitations. First, it is important to note that the clinical data analyzed in this study were retrospective in nature, potentially introducing inherent bias in the collected data. Second, as our study is retrospective, the reliability of the nomogram requires additional confirmation through prospective research. Additionally, since this study was carried out at a single center, it is essential to validate externally by leveraging data from diverse centers. Third, while our predictive model has clinical practicality, this study lacks innovative research indicators for predicting the risk of apnea, such as gestational smoking, birth weight, gestational age, maternal pregnancy status, feeding methods, perinatal infections, antibiotic use, and body mass index. Therefore, we intend to conduct a prospective cohort study to enhance the predictive accuracy of future apnea prediction models.

In summary, this study constructed a nomogram model to predict apnea in bronchiolitis based on underlying diseases, feeding difficulties, tachypnea, WBC, and pulmonary consolidation. Applying this model to predict the risk of apnea in bronchiolitis in clinical practice is beneficial for formulating more optimized clinical treatment plans. High-risk patients can be identified early and monitored closely for improved clinical prognosis.

## Data Availability

The original contributions presented in the study are included in the article/Supplementary Material, further inquiries can be directed to the corresponding authors.
